# Adipocyte Septin-7 attenuates obesogenic adipogenesis and promotes lipolysis to prevent obesity

**DOI:** 10.1016/j.molmet.2025.102114

**Published:** 2025-02-25

**Authors:** Liran Xu, Chao Yang, Kaidan Pang, Ying Zhang, Yu He, Siyu Liu, Huijing Tian, Zehua Shao, Siyu Wang, Xingqian Liu, Ting Li, Yapeng Cao, Luqin Yan, Jinjin Liu, Yanan Wang, Yongxin Li, Wei Zhao, Youhua Wang, Yang Yan, Shengpeng Wang

**Affiliations:** 1 Department of Pharmacology, School of Basic Medical Sciences, Xi'an Jiaotong University, Xi'an, Shaanxi, China; 2 Department of Cardiovascular Surgery, The First Affiliated Hospital of Xi'an Jiaotong University, Xi'an, 710000, China; 3 Department of Cardiovascular Medicine, The First Affiliated Hospital of Xi'an Jiaotong University, Xi'an, 710000, China; 4 Patient Service Center, The First Affiliated Hospital of Xi'an Jiaotong University, Xi'an, 710000, China; 5 Med-X institute, Center for Immunological and Metabolic Diseases, and Department of Endocrinology, The First Affiliated Hospital of Xi'an Jiaotong University, Xi'an, 710000, China; 6 Department of General Surgery, The First Affiliated Hospital of Xi'an Jiaotong University, Xi'an, 710000, China; 7 Institute of Sports and Exercise Biology, School of Physical Education, Shaanxi Normal University, Xi'an, 710000, China

**Keywords:** Septin-7, Obesity, Adipocyte, Adipogenesis

## Abstract

**Objectives:**

The white adipose tissue (WAT) expansion plays a significant role in the development of obesity. Cytoskeletal remodeling directly impacts adipogenic program, however, the precise mechanism remains poorly understood. Here, we identified a crucial role of Septin-7 (SEPT7), a cytoskeleton component, in the regulation of diet-induced processes of adipogenesis, lipogenesis, and lipolysis in WAT.

**Methods:**

A high-fat diet (HFD)-induced obesity model was constructed using mice with inducible adipocyte-specific SEPT7 deficiency. The impact of SEPT7 on adipocyte morphology, cell number and metabolism capacity were evaluated with immunofluorescence, isoproterenol induced lipolysis assay, glucose tolerance test and insulin tolerance test. Adipocyte mTmG reporter line was established to trace *in vivo* adipogenesis. The preadipocyte 3T3-L1 cell was induced for exploring role of SEPT7 in adipocyte differentiation. qRT-PCR and Western-blot were used to investigate the expression of PPARγ, C/EBPα, and HSL in 3T3-L1 cell with siRNA-mediated SEPT7 knockdown.

**Results:**

SEPT7 expression was greatly induced in obesogenic human and murine adipocytes. Mice lacking SEPT7 in mature white adipocytes demonstrated defective differentiation of preadipocyte into mature adipocytes when fed HFD resulting in larger adipocytes, increased WAT inflammation and reduced lipolysis, which leading to increased WAT mass, liver fat accumulation and impaired glucose tolerance. Mechanistically, we identified SEPT7 restrains store-operated Ca^2+^ entry (SOCE) and regulates adipocyte adipogenesis and lipolysis by targeting PPARγ, C/EBPα and HSL.

**Conclusions:**

We demonstrated that SEPT7 negatively regulates adipogenesis while promotes lipolysis and its repression drives WAT expansion and impaired metabolic health.

## Highlights

• Septin-7 (SEPT7) plays a crucial role in regulating diet-induced obesity.
• SEPT7 deficiency leads to larger adipocytes, increased WAT inflammation and impaired glucose tolerance in obese mice.
• SEPT7 negatively regulates adipogenesis while promoting lipolysis.

## 1 Introduction

Obesity maintains a significant public health concern [1]. Shifts in lifestyle have accompanied economic development and social change, resulting in a gradual increase in the prevalence of obesity [2]. Epidemiological studies have demonstrated a correlation between obesity and an increased risk of developing a number of chronic diseases, including cardiovascular disease, cerebrovascular disease, diabetes and certain types of cancer [[3], [4], [5], [6]]. The ongoing increase in prevalence and the associated health and economic consequences impose a significant burden on healthcare systems [7].

The defining pathological alteration associated with obesity is an increase in the quantity of adipose tissue within the body [8]. This expansion of the adipose tissue in obesity may occur as a response to all sorts of stress, be it metabolic, mechanical or inflammatory [9]. During this process, the increase of adipocytes in both size and number correlates with cytoskeletal remodeling, as cytoskeletal remodeling plays a crucial role in adipocyte differentiation and lipid droplet (LD) enlargement [10,11]. Actomyosin, a cytoskeletal component, is critical for the acute induction of oxidative metabolism in adipocytes [12]. A drastic increase in filamentous (F)-actin, another cytoskeletal component, was found associated with diet-induced expansion of primary adipocytes as well [13].

Septin proteins are integral components of the cytoskeleton, exerting significant regulatory influences on the processes of cell division and cell polarity [14]. The family is comprised of four subgroups and 13 members, whose functions are closely related to their central roles in the cytoskeleton [15]. These include roles as molecular scaffolds, cell membrane barriers, regulators of cell mobility, apoptosis, endocytosis, cytokinesis, and mechanosignaling, among others [[16], [17], [18], [19], [20], [21], [22]]. Some of the septin family members shows potential in modulating fat metabolism. SEPT9 has been revealed as a regulator of LD behavior [23]. Adipocyte SEPT11 elevates in obesity individual, and functions as a mediator in insulin-induced fat accumulation [24].

SEPT7 is a prominent member of this family with a distinctive structural configuration that exists as a monomer within the complex and is capable of attaching to the extremities of hexameric building blocks, thereby facilitating the formation of non-polarized filaments [25]. Recent studies have demonstrated that SEPT7 plays a pivotal role in the nervous system and reproductive system, and has been associated with the pathogenesis of cancers including gliomas, papillary thyroid carcinoma, and hepatocellular carcinoma [26,27]. Cheng et al. found that overexpression of SEPT7 could promote the differentiation of bovine intramuscular preadipocyte [28]. Nevertheless, the precise function of SEPT7 in obesity remains largely undiscovered.

A recent study on single-cell mapping of human and mice white adipose tissue revealed an overexpression of SEPT7 in human with higher BMI as well as mice fed a high-fat diet (HFD) [29]. In this study, we found adipocyte-specific SEPT7 deficiency promoted hypertrophy of white adipocyte and increased the number of adipocyte following 16 weeks of HFD. A comprehensive examination of glucose and fat metabolism in this obese mouse model substantiated that adipocyte SEPT7 deletion resulted in a reduction in body glucose tolerance, leading to liver fat accumulation and elevated serum triglyceride levels. Further studies have demonstrated that SEPT7 can influence the accumulation of adipose tissue and the development of obesity by regulating the expression of genes such as PPARγ, C/EBPα and HSL, which are involved in the processes of adipogenesis, lipogenesis and lipolysis in adipocytes. The evidence presented here suggests that SEPT7 plays a significant role in the development of obesity and represents a promising new therapeutic target for the intervention of obesity.

## 2 Methods and materials

### 2.1 Generation of transgenic mice and establishment of obesity model

All mice were backcrossed onto the C57BL/6 background for at least 8 generations. Experiments were performed with Cre-negative littermates as controls. Male and female animals at an age of 8–12 weeks were used if not stated otherwise. Mice were housed under a 12-hour light–dark cycle with free access to food and water and under specific pathogen-free conditions. All mouse experiments were performed in accordance with the guidelines of the institutional animal care & use committee of Xi'an Jiaotong University.

The tamoxifen-induced adipocyte-specific SEPT7 deletion mouse model was developed by crossing Adipoq-CreER^T2^ line and SEPT7^fl/fl^ line. Adipoq-CreER^T2^ mice that allow for tamoxifen-dependent adipocyte-specific Cre activation have been described before [30]. Sept7-flox mice (SEPT7^fl/fl^) were generated in C57BL/6S line by Shanghai Model Organisms Center, Inc. (SMOC). Briefly, Exon 4 of Sept7 gene (ENSMUST00000165594) was modified by flox using the principle of homologous recombination in fertilized egg. Cas9 mRNA and gRNA were obtained by in vitro transcription; a donor vector containing 5′ homologous arm, flox region, and 3′ homologous arm was constructed by In-Fusion cloning. Cas9 mRNA, gRNA and donor vector were microinjected into the fertilized eggs of C57BL/6J mice to obtain F0 generation mice. Adipoq-CreER^T2^ allows tamoxifen-inducible Cre expression in adiponectin-positive cells. Adiponectin is a protein encoded by the Adipoq gene and is produced primarily in adipose tissue. SEPT7^fl/fl^ allows the complete deletion of SEPT7 gene on double-chain DNA by the Cre recombinase.

The Adipoq-CreERT2; mT/mG, SEPT7^fl/fl^ mice were generated by crossing Adipoq-CreERT2; SEPT7^fl/fl^ line with mT/mG reporter line [9,31].

In diet-induced obesity experiment, 24 mice were allocated to HFD group and CD group (as control) equally through simple randomization, then fed designated diet for 16 weeks and killed for fat tissue collection and adipocyte isolation, or whole mount tissue staining. Both groups are housed in the same environment concerning temperature, moisture, ventilation, illumination, drinking water and hygiene.

### 2.2 Histological analysis

Harvested adipose tissue was stationed on glass slide, fixed in Carnoy's fixative for 2–4 h at room temperature. Then it was washed in 70% EtOH for 15 min, rinsed in distilled water for 5 min and stained in H&E (Cat#ST047, Hete& Cat#E8090, Solarbio). Then it was washed in 70% EtOH 15 min, in 95% EtOH 15 min, in 100% EtOH 15 min, and then cleared in xylene and mounted with Permount.

Bright-field images were taken with Olympus microscope. Digitized images were analyzed by Image-Pro Plus 6.0 software (Media Cybernetics).

BODIPY staining. Differentiated 3T3-L1 cells were fixed with 2% formaldehyde and 0.2% glutaraldehyde in PBS for 15 min and then washed 3 times in PBS for 10 min. For BODIPY staining, fixed cells were blocked in 2% BSA in PBS with 0.3% Triton X-100 for 1 h and incubated with BODIPY (Invitrogen, catalog number D3922, 1:1000) and DAPI (Invitrogen, catalog number D1306, 1:1000) for 1 h at room temperature. Cells were washed with PBS, followed by imaging with Leica TCS SP5 confocal microscope.

### 2.3 Cell culture

3T3-L1 adipocytes were obtained from Procell, Inc. (Cat#CL-0006) and cultured with DMEM (Gibco) supplemented with 10% fetal bovine serum (FBS) (BI) and 1% penicillin and streptomycin.

3T3-L1 cells was kept 2 days after reaching confluency in DMEM containing 1 μg/ml insulin (Gibco), to induce differentiation of adipocyte precursor cells. Conditioned medium was given to the 3T3-L1 cells 2 and 4 days after cells had reached confluency. Thereafter cells were cultured for an additional 6 days in DMEM supplemented 5 μg/ml insulin with medium changes every 2 days.

### 2.4 Small interfering RNA (siRNA)-mediated knockdown

Cells were transfected with siRNAs using Opti-MEM (ThermoFisher) and Lipofectamine RNAiMAX (Invitrogen) as described previously. For transfection of cells, 20 pmol of siRNA were mixed gently with RNAiMAX, and incubated for 30 min at room temperature. The mixture was then added to cell culture medium. The medium was changed to fresh complete medium after 6 h. Experiments were performed 48 h later. siRNAs against SEPT7 were from Genepharma. The targeted sequences of mouse siRNAs directed against RNAs encoding SEPT7 was as follows: 5′-AAUGAUAGUAUUACUACCU-3′, 5′-AGGUAGUAAUACUAUCAUU-3′.

### 2.5 qRT-PCR analysis

Total RNA was isolated from cells or tissues with Trizol reagent (Cat#15596018, Invitrogen, Grand Island, NY). 1 μg of RNA was used for cDNA synthesis with the cDNA synthesis kit (Cat#170-8891, BioRad) according to the manufacturer's instructions. qRT-PCR was performed with universal SYBR green mix (Cat#172-5122, BioRad) on the StepOne Plus System (Applied Biosystems, Grand Island, NY). 2^-△△CT^ method was used to quantify the relative expression of target genes. Relative expression levels were obtained by normalization with 18S values. qRT-PCR primer sequences were as follows (5′-3′):

18S F: GGCCCTGTAATTGGAATGAGTC; R: CCAAGATCCAACTACGAGCTT

SEPT7 F: GAAGGTGGTGTTCAGTTGCTGC; R: GCATCTGACGTCTGTTCACTCG

C/EBPα F: CGCTGGTGATCAAACAAGAG; R: GGTGGCTGGTAGGGGAAG

PPARγ F: ACGCGAGGAGGTCAAGAAG; R: TCAATGGGAGTTAAGAAGAATTT

HSL F: CCACGAGCCCTACCTCAAGAA; R: GCATATCCGCTCTCCAGTTGAA

CD36 F: TTGAAAAGTCTCGGACATTGAG; R: TCAGATCCGAACACAGCGTA

Dgat2 F: TCATGGGTGTCTGTGGGTTA; R: CAGAGTGAAACCAGCCAACA

Perilipin F: GGACTTACAAACAGCAACAGACC; R: CATCTGTGAGTTGGTGGACACT

Glut1 F: GGATCCCAGCAGCAAGAAG; R: CCAGTGTTATAGCCGAACTGC

Glut4 F: GACGGACACTCCATCTGTTG; R: GCCACGATGGAGACATAGC

Fabp4 F: GGATGGAAAGTCGACCACAA; R: TGGAAGTCACGCCTTTCATA

Egr1 F: GTCCTTTTCTGACATCGCTCTGA; R: CGAGTCGTTTGGCTGGGATA

Adrb1 F: GCTGATCTGGTCATGGGATT; R: CACACAGGGTCTCAATGCTG

Adrb2 F: CTTGAAAGAGCACAAAGCCC; R: GTTGACGTAGCCCAACCAGT

Adrb3 F: GGCAGTGCAGGAGGAAGATG; R: CTGGCAGAACCTGAGGCAAC

Mgl F: GACGGACAGTACCTCTTTTG; R: AGAAAAGTGGTTGGCCTCT

### 2.6 Whole mount staining

For cryosections, tissue was embedded in optimal cutting temperature compound (‘OCT’, Tissue-Tek, Cat#4583), cryosectioned at −20 °C (5 μm sections), and transferred onto adhesive slides. For cell samples, cells were fixed with 4% PFA for 15 min. Tissue or cell slides were blocked with 5% bovine serum albumin (Cat#V900933, Sigma) and permeabilized with 0.3% Triton X-100(Cat#T8200, Solarbio) for 1 h at room temperature. Samples were stained with antibodies against PPARγ (1:100, Cat#2443S, CST), C/EBPα (1:100, Cat#29388-1-AP, Proteintech), HSL (1:100, Cat#4107S, CST), SEPT7 (1:100, Cat#13818-1-AP, Proteintech), overnight at 4 °C. The next day, slides were incubated with corresponding AlexaFluor conjugated isotype-specific secondary antibodies (1:200, Invitrogen) at room temperature for 1 h. Nuclei were counterstained with DAPI (1 μg/mL, Thermo Fisher Scientific) for 5 min at room temperature. After washing three times with PBS, the samples were mounted with mounting media (Vector Laboratories). Immunofluorescence imaging was performed using a Leica TCS SP5 confocal microscope.

### 2.7 Western blot

Sample were lysed by RIPA buffer (Cell Signaling, Cat#9806) supplemented with protease and phosphatase inhibitors (Cat#04906837001, Roche). Lysates were centrifuged at 12,000 rpm at 4 °C for 10 min. Supernatants were then subjected to SDS-PAGE and transferred to PVDF membranes. After blocking in 5% skim milk for 1 h, blots were probed using the below primary antibodies: GAPDH (1:1000, Cat#60004-1-Ig, Proteintech), PPARγ (1:1000, Cat#2443S, CST), C/EBPα (1:1000, Cat#29388-1-AP, Proteintech), HSL (1:1000, Cat#4107S, CST), SEPT7 (1:1000, Cat#13818-1-AP, Proteintech) were used as the loading control. Blots were developed using the ECL detection system (BioRad).

### 2.8 Glucose and insulin tolerance test (GTT & ITT)

After overnight fasting glucose was administered to mice intraperitoneally at a dose of 2 mg/g body weight when on CD and of 2.75 mg/g body weight when on HFD. Blood samples were taken from the tail vein before and after glucose application at indicated time points, and glucose levels were measured using a glucometer (Accu-Chek Aviva). For ITT, mice were injected intraperitoneally with insulin (0.75 mU/g), and glucose levels were determined as described above.

### 2.9 Isoproterenol induced lipolysis assay

Adipocyte buffer was added to an equal amount of adipocyte suspension, diluted to a total volume of 250 μL, and transferred to a new EP tube. Added isoproterenol (ISO) to reach the final concentration of 10 nmol/L, and the resulting adipocyte suspension was incubated in an incubator at 37 °C for 2 h. The incubated adipocyte suspension was heated in a 70 °C water bath for 10 min, centrifuged at 2000 rpm for 5 min at room temperature, and then the supernatant was taken, and a spectrophotometer and a standard curve could be used to determine the glycerol and fatty acid content.

### 2.10 Quantification of total cholesterol, triglyceride, HDL-C and LDL-C

Mice serum were separated by centrifuging blood sample at 3000 rpm for 20 min at room temperature. The liver homogenate was centrifuged at 2500 rpm for 10 min at 4 °C and the supernatant was collected. Mix the sample with detection reagents provided in the total cholesterol assay kit (A111-1-1, Nanjing Jiancheng). Draw a standard curve with the total cholesterol concentration of the calibrator as the horizontal axis and the absorbance at 500 nm as the vertical axis. Substitute the absorbance value measured by the sample into the standard curve to obtain the total cholesterol concentration of the sample. The quantification of triglyceride, HDL-C and LDL-C are similar, with different detection kit (A110-1-1, A112-1-1, A113-1-1, Nanjing Jiancheng) and different max absorbance wavelength at 510 nm, 600 nm and 600 nm respectively.

### 2.11 Metabolic cage assay

Adipocyte-specific SEPT7 deletion mice and control mice (all male) were fed a high-fat diet for 8 weeks. After being housed in single cages for 1 week, all mice were set in a Promethion Metabolic Cage (Sable Systems International) for 24 h (12-hour day/night cycle). Animal activity was measured using Promethion XYZ Beambreak Activity Monitor. Food and water intake, movement distance, VO2, and VCO2 were measured using Promethion precision sensors. Energy expenditure was calculated in kilocalories/hour by utilizing the Weir equation. Respiratory exchange ratio equals VCO2 divided by VO2 [32]. At the end of the experiment, all mice were sent into the nuclear magnetic resonance spectroscopy to decide their fat mass and lean mass. O_2_ consumption, CO_2_ production and energy expenditure were adjusted by individual lean mass.

### 2.12 Store-operated Ca^2+^ entry (SOCE) assay

SOCE was measured in cells loaded with Fluo-4 in an 8 chambered coverglass system (Cellvis) by confocal microscopy (Leica TCS SP5). Cells were initially imaged in the presence of 1.2 mmol/L external calcium (high calcium) and sequentially exposed to 1.3 mmol/L EGTA (no calcium) with 1.25 μmol/L thapsigargin to empty endoplasmic reticulum calcium stores and 2 mmol/L free external calcium (calcium addback) [33]. To inhibit SOCE, cells were treated with 10 μmol/L BTP2 (selective SOCE inhibitor, Selleck) for interventions.

### 2.13 Statistical analysis

All statistical analyses were performed with GraphPad Prism 9. Trial experiments or experiments done previously were used to determine sample size with adequate statistical power. Samples were excluded in cases where RNA/cDNA quality or tissue quality after processing was poor (below commonly accepted standards). Animals were excluded from experiments if they showed any signs of sickness. The investigator was blinded to the group allocation during the experiment. Data are presented as mean ± SEM. Comparisons between 2 groups were performed with unpaired 2-tailed Student's t test (both groups passed normality test with same SD), Welch's t-test (both groups passed normality test without same SD) or the Mann–Whitney U test (for nonparametric tests), P ≤ 0.05 was considered to be statistically significant.

### 2.14 Study approval

Studies using animals and biological samples were approved by the ethics committee of Xi'an Jiaotong University and conform to the guidelines of the 2000 Helsinki declaration.

## 3 Results

### 3.1 SEPT7 expression is induced in obesogenic human and murine adipocytes

**Figure 1:**
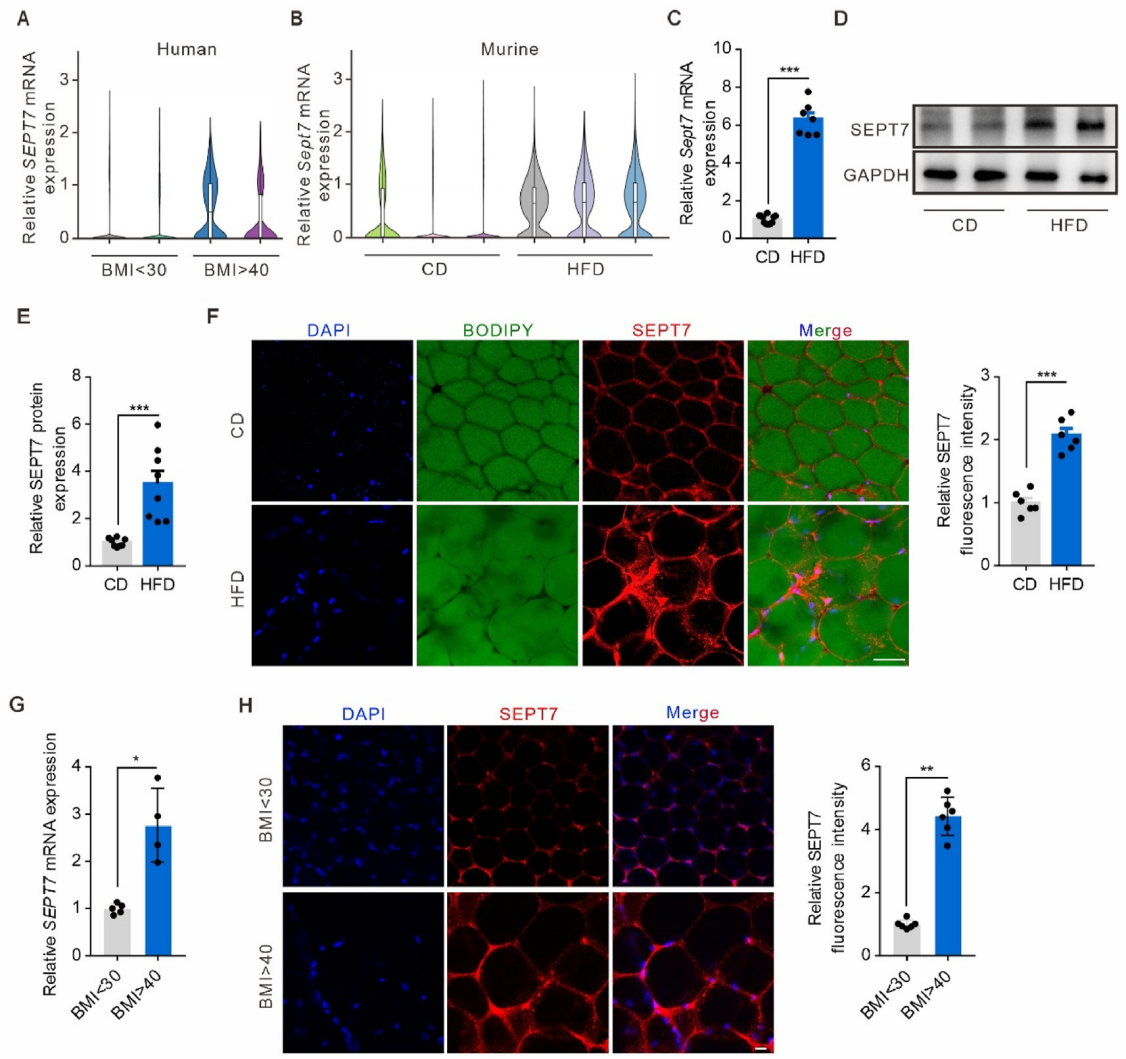
**SEPT7 is highly induced in obesogenic WAT**. (**A**) Single-cell sequencing data showing the adipocyte expression of SEPT7 in human with BMI<30 or >40. (**B**) Single-cell sequencing data showing the adipocyte expression of SEPT7 in mice on CD or HFD. (**C**) qRT-PCR showing mRNA expression of SEPT7 in adipose tissue isolated from mice fed CD or HFD for 16 weeks (n = 7–12). (**D-E**) Western blot analysis (**D**) and quantification (**E**) of SEPT7 protein levels in adipose tissue isolated from mice fed CD or HFD for 16 weeks (n = 8). (**F**) Representative images (left) and quantifications (right) of immunofluorescence staining for SEPT7(red), BODIPY(green) and DAPI(blue) in adipose tissue isolated from mice fed CD or HFD for 16 weeks (n = 6). Scale bar, 50 μm. (**G**) qRT-PCR showing mRNA expression of SEPT7 in adipose tissue isolated from human with BMI<30 or >40 (n = 4–5). (**H**) Representative images (left) and quantifications (right) of immunofluorescence staining for SEPT7(red) and DAPI(blue) in adipose tissue isolated from human with BMI<30 or >40 (n = 6). Scale bar, 20 μm. Shown are mean values ± SEM. ∗P ≤ 0.05; ∗∗P ≤ 0.01; ∗∗∗P ≤ 0.001.

To investigate the specific targets of adipocyte regulation of metabolism in obese individuals, a single-cell sequencing database of adipocytes from obese human and mouse was analyzed [29]. SEPT7, a member of the septin family, was identified through a differential gene expression (DEG) comparison in both human and mice (Figure 1A,B, Supplementary Fig. 1A&D). This comparison showed that SEPT7 expression in adipocyte is higher in human with BMI>40 than those with BMI<30. This tendency remained the same in the mice, as HFD-fed mice exhibited a higher expression of adipocyte SEPT7 than CD (chow diet)-fed controls (Figure 1A, B). To corroborate these findings, an obese mouse model was created by administering a 16-week HFD or CD to 8-week-old C57 mice (Supplementary Figure. 1B, C), and adipose tissue SEPT7 expression was analyzed. qRT-PCR (Figure 1C) and Western blot assays (Figure 1D, E) indicated a substantial upregulation of SEPT7 at both the transcriptional and protein levels in the adipocyte of HFD mice. Immunofluorescence demonstrated a notable elevation in SEPT7 expression within the adipocytes of HFD mice in comparison to controls (Figure 1F). To further validate the SEPT7 expression in obesity, we obtained fat samples from obese human. Both qPCR and whole mount staining showed that SEPT7 expression was increased in adipose tissue from obese individuals with BMI>40 compared with the BMI<30 group (Figure 1G–H). These findings confirmed that SEPT7 expression is indeed augmented in obesogenic adipocytes.

### 3.2 Adipocyte SEPT7 deletion promotes diet-induced obesity development

**Figure 2:**
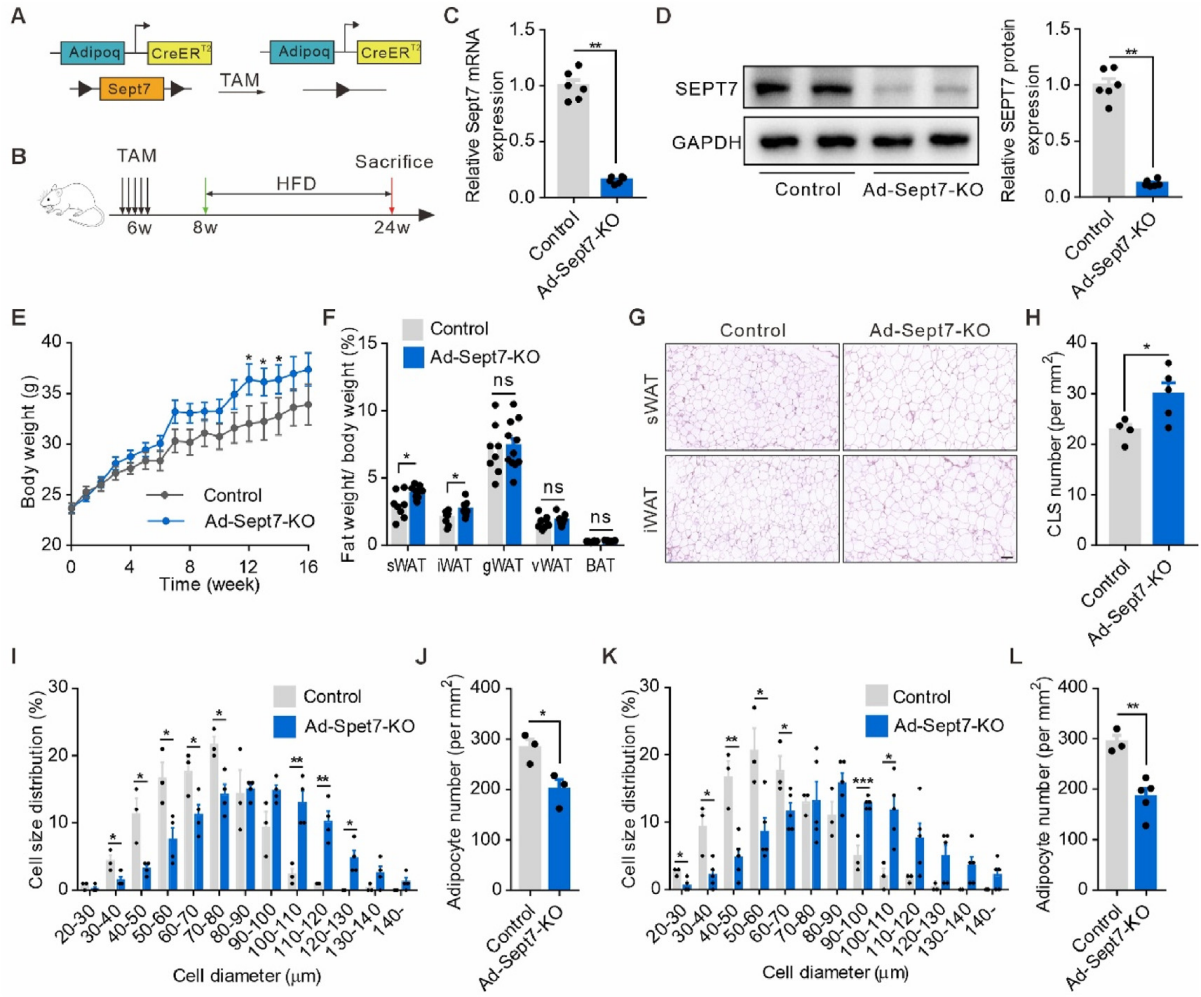
**Adipocyte-specific SEPT7 deletion promotes diet-induced WAT expansion**. (**A**) Schematic representation showing the genetic strategy for the generation of the Adipoq-CreER^T2^; SEPT7^fl/fl^ mice. (**B**) Schematic diagram of the experimental design. Adipoq-CreER^T2^; SEPT7^fl/fl^ mice were injected with tamoxifen on 5 consecutive days before 16-week HFD. (**C**) qRT-PCR showing mRNA expression of SEPT7 in adipose tissue isolated from Control and Ad-*Sept7*-KO mice fed HFD for 16 weeks (n = 6). (D) Western blot analysis (left) and quantification (right) of SEPT7 protein levels in adipose tissue isolated from Control and Ad-Sept7-KO mice fed HFD for 16 weeks (n = 6). (**E**) Body weight of Control and Ad-Sept7-KO mice fed HFD for 16 weeks (n = 6–8). (**F**) Various fat weight/body weight ratio from Control and Ad-*Sept7*-KO mice fed HFD for 16 weeks (n = 8–11). (**G**) H&E-stained sWAT and iWAT sections from Control and Ad-Sept7-KO mice after 16 weeks of HFD. Scale bar, 100 μm. (**H**) Number of CLS per square millimeter of iWAT in (**G**) (n = 4–5). (**I**) Distribution of size of sWAT adipocytes prepared from control and Ad-Sept7-KO mice fed HFD for 16 weeks (n = 3–4). (**J**) Adipocyte number per square millimeter of sWAT from control and Ad-Sept7-KO mice (n = 3). (**K**) Distribution of size of iWAT adipocytes prepared from control and Ad-Sept7-KO mice fed HFD for 16 weeks (n = 3–5). (**L**) Adipocyte number per square millimeter of iWAT from control and Ad-Sept7-KO mice (n = 3–5). Shown are mean values ± SEM. ∗P ≤ 0.05; ∗∗P ≤ 0.01; ∗∗∗P ≤ 0.001; ns, not significant; Control, Adipoq-CreER^T2^(-); SEPT7^fl/fl^ mice. Ad-*Sept7*-KO, Adipoq-CreER^T2^(+); SEPT7^fl/fl^ mice. sWAT, subcutaneous white adipose tissue. iWAT, inguinal white adipose tissue. gWAT, gonadal white adipose tissue. vWAT, visceral white adipose tissue. BAT, Brown adipose tissue.

To further elucidate the precise role of SEPT7 in the adipogenic processes, a tamoxifen-induced adipocyte-specific Septin7-deficient mice was constructed by crossing Adipoq-CreER^T2^ line with SEPT7^fl/fl^ line (Figure 2A). Tamoxifen was induced in 6-week-old transgenic mice (marked as Adipoq-CreER^T2^(+); SEPT7^fl/fl^ or Ad-*Sept7*-KO) and the control group (marked as Adipoq-CreER^T2^(-); SEPT7 ^fl/fl^) (Figure 2B). qRT-PCR and Western blot assays demonstrated that SEPT7 expression was markedly diminished in the adipocyte of Ad-*Sept7*-KO mice (Figure 2C, D). Under CD, there was no significant difference in body weight and fat mass between the Ad-*Sept7*-KO mice and the control group (Supplementary Figure 2A–D). Following 16-weeks HFD, the male Ad-*Sept7*-KO mice demonstrated a greater increase in body weight compared to the control group (Figure 2E). The mass of gonadal white adipose tissue (gWAT), visceral white adipose tissue (vWAT) and brown adipose tissue (BAT) were not different between the two strains, but the mass of the subcutaneous white adipose tissue (sWAT) and inguinal white adipose tissue (iWAT) increased significantly in the Ad-*Sept7*-KO mice compared with the control (Figure 2F). H&E staining revealed a significant increase in the size of sWAT and iWAT adipocytes in the Ad-*Sept7*-KO mice compared with the control (Figure 2G, I-L). Analysis of iWAT from HFD-fed Ad-*Sept7*-KO mice revealed increased crown-like structures (CLS), indicating a pro-inflammatory signature induced by adipocyte SEPT7 deletion (Figure 2H). However, the female Ad-*Sept7*-KO mice had similar body weight gain compared to female wild type mice after HFD feeding (Supplementary Figure 3A–D). Of note, SPET7 expression in adipose tissue was higher in female mice compared to male mice (Supplementary Figure 3E–F). These findings indicated that adipocyte-specific SEPT7 deficiency promoted diet-induced adiposity in male mice.

As BAT has a pivotal role in energy metabolism and the BAT mass is not altered by SEPT7, we conducted the metabolic cage experiment to evaluate the effect of SEPT7 deletion on mice metabolism. We designed a metabolic cage experiment to evaluate the energy expenditure levels of inducible SEPT7 adipocyte-specific deletion mice and control mice after 8 weeks of high-fat diet. Mice are placed in gas-tight metabolic cages through which a flow of fresh air is passed. The system collects and mixes the expired air, measures the flow rate and analyzes the gas concentration of the incoming and outgoing air for both O2 and CO2 [34]. Energy expenditure is measured by calculating the calories using Weir's equation [35]. The results showed that after high-fat diet feeding, the total body weight and fat mass of the SEPT7 deletion group mice were significantly higher than those of the control group, and there was no significant difference in lean mass between the two groups (Supplementary Figure 4A). After metabolic cage evaluation, there were no significant differences in oxygen consumption, carbon dioxide production, respiratory exchange ratio, energy expenditure, locomotor activity and cumulative food intake between the two groups of mice (Supplementary Figure 4B–I, L). The pedestrian locomotion, total distance in cage and cumulative water intake of SEPT7 deletion mice were all lower than those of the control group (Supplementary Figure 4J, K, M). We consider that these differences are due to the SEPT7-deletion-induced obesity, and SEPT7 has no clear effect on the energy expenditure of mice.

Altogether, these findings indicated that adipocyte-specific SEPT7 deficiency promoted diet-induced adiposity in male mice.

### 3.3 Adipocyte SEPT7 suppresses obesogenic adipogenesis

**Figure 3:**
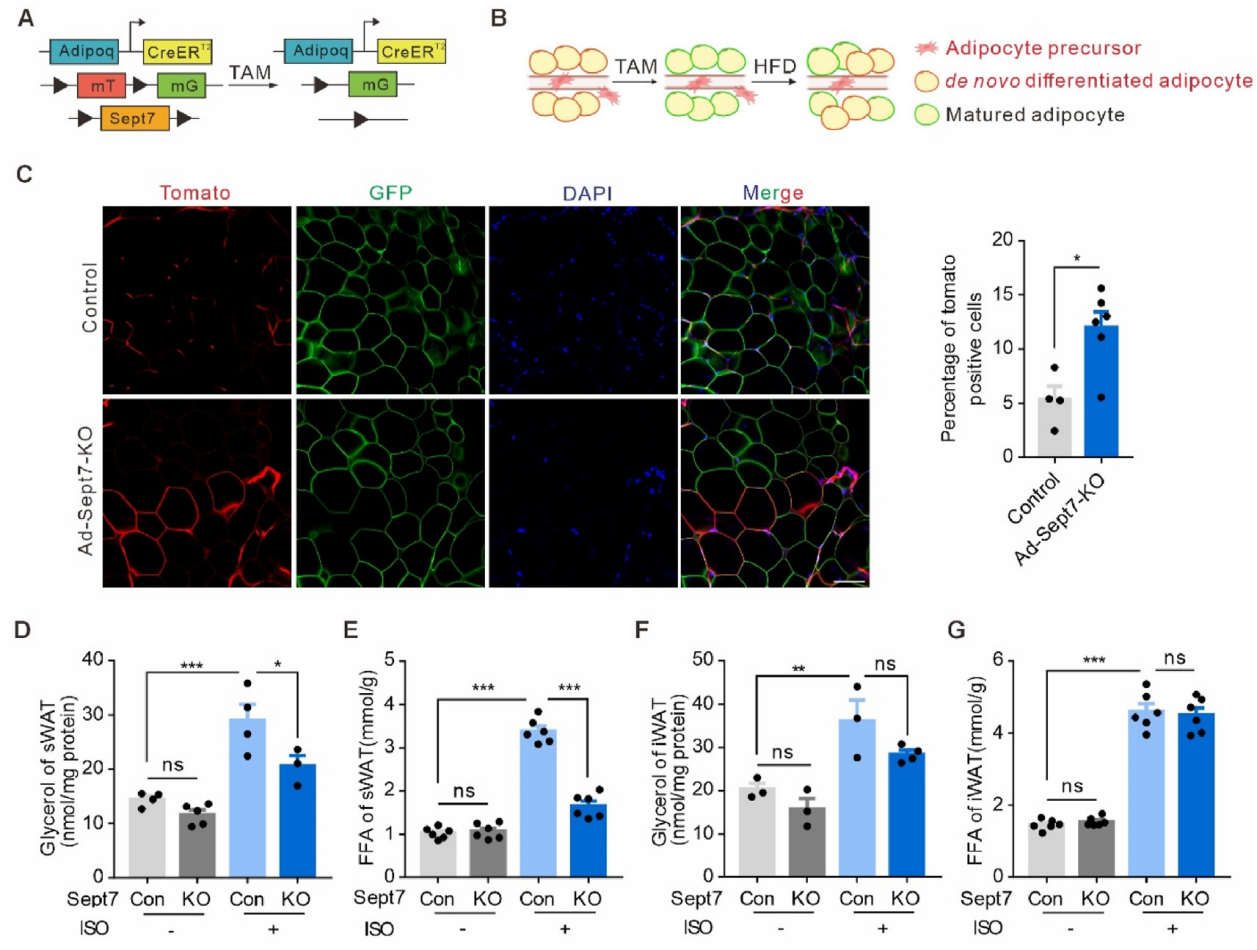
**Adipocyte-specific SEPT7 deletion promots obesogenic adipogenesis**. (**A**) Schematic representation showing the genetic strategy for the generation of the Adipoq-CreERT2; mT/mG; SEPT7^fl/fl^ mice. (**B**) Experimental design of analysis of adipogenesis *in vivo* using Adipoq-CreERT2; mT/mG mice. (**C**) Statistical analysis and representative images of adipocyte tracing in Adipoq-CreERT2; mT/mG; SEPT7^fl/fl^ (Ad-Sept7-KO) and Adipoq-CreERT2; mT/mG; mice (Control) after tamoxifen treatment and 16 weeks of HFD in iWAT (n = 4–6); Scale bar, 50 μm(**D**) Quantification of glycerol in isoproterenol induced lipolysis of sWAT from control and Ad-Sept7-KO mice on HFD (n = 3–5) (**E**) Quantification of FFA in isoproterenol induced lipolysis of sWAT from control and Ad-Sept7-KO mice on HFD (n = 6). (**F**) Quantification of glycerol in isoproterenol induced lipolysis of iWAT from control and Ad-Sept7-KO mice on HFD (n = 3–4). (**G**) Quantification of FFA in isoproterenol induced lipolysis of iWAT from control and Ad-Sept7-KO mice on HFD (n = 6). Shown are mean values ± SEM. ∗P ≤ 0.05; ∗∗P ≤ 0.01; ∗∗∗P ≤ 0.001; ns, not significant.

The increased weight gains and total adipocyte number in WAT from HFD-fed Ad-*Sept7*-KO mice suggested an increase in adipocyte adipogenesis. To assess the *in vivo* role of SEPT7 in adipocyte hyperplasia in response to HFD feeding, we performed tamoxifen pulse-chase experiments in adipocyte-specific, tamoxifen-inducible Cre transgenic mice (Adipoq-CreERT2) crossed with the SEPT7^flox/flox^ and mT/mG reporter line, which switches from membrane-targeted Tomato expression to membrane-targeted GFP expression upon Cre-mediated recombination (Figure 3A–B). One week after induction of Cre activity, mice were fed with 16 weeks HFD. iWAT from wild-type mice contained ∼6% mTomato-positive adipocytes, which formed from mTomato-positive adipocyte precursor cells after tamoxifen treatment. Interestingly, the number of mTomato-positive cells was increased by ∼2 fold in Ad-*Sept7*-KO mice (Figure 3C). Thus, adipocyte-specific SEPT7 deletion promoted adipogenesis by facilitating the generation of *de novo* adipocyte recruitment from adipocyte precursors (adipogenesis). We next studied whether the functional relevance of SEPT7 in adipocyte hypertrophy is due to reduced adipocyte lipolysis. While the basal lipolytic activity is comparable between wild-type and SEPT7-KO adipocyte, the isoproterenol-induced glycerol and free fatty acid (FFA) release were dramatically inhibited in adipocyte from Ad-*Sept7*-KO mice (Figure 3D–G), suggesting a reduced lipolysis activity in SEPT7 deficient adipocyte.

### 3.4 Adipocyte-specific SEPT7 deletion impaired glucose tolerance and hepatic fat metabolism

**Figure 4:**
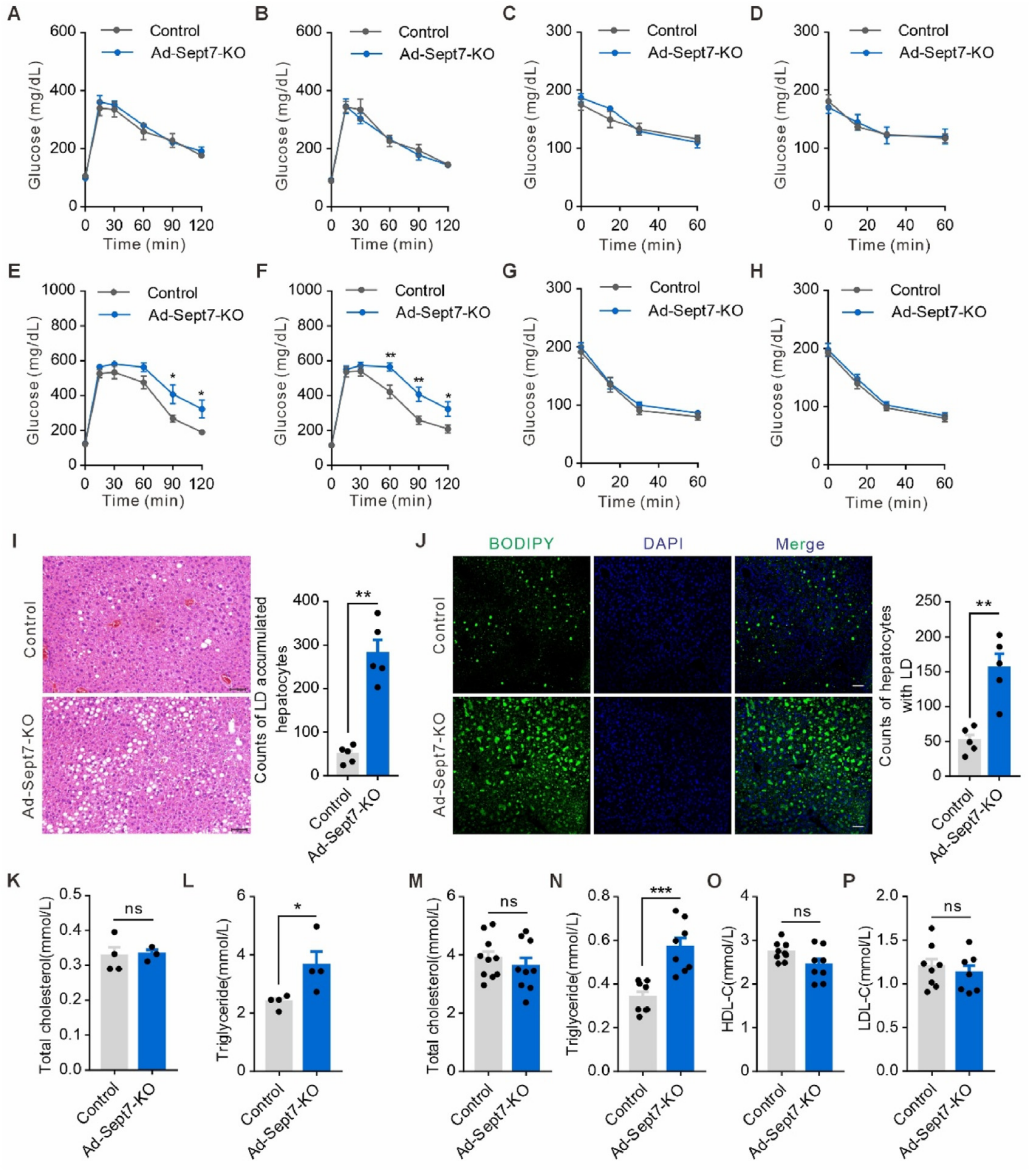
**Adipocyte-specific SEPT7 deletion impairs glucose tolerance and hepatic fat metabolism**. (**A**) Glucose tolerance in control and Ad-Sept7-KO mice fed a CD for 8 weeks (n = 3–6). (**B**) Glucose tolerance in control and Ad-Sept7-KO mice fed a CD for 16 weeks (n = 4). (**C**) Insulin tolerance in control and Ad-Sept7-KO mice fed a CD for 8 weeks (n = 3–5). (**D**) Insulin tolerance in control and Ad-Sept7-KO mice fed a CD for 16 weeks (n = 4). (**E**) Glucose tolerance in control and Ad-Sept7-KO mice fed a HFD for 8 weeks (n = 11). (**F**) Glucose tolerance in control and Ad-Sept7-KO mice fed a HFD for 16 weeks (n = 9–11). (**G**) Insulin tolerance in control and Ad-Sept7-KO mice fed a HFD for 8 weeks (n = 8–10). (**H**) Insulin tolerance in control and Ad-Sept7-KO mice fed a HFD for 16 weeks (n = 10–11). (**I**) (left) H&E-stained liver sections from control and Ad-Sept7-KO mice fed a HFD for 16 weeks. Scale bar, 50 μm.(right) the number of adipercytes in liver per scope view (n = 5). (**J**) Representative images and statistical analysis of BODIPY staining in liver of control and Ad-Sept7-KO mice fed a HFD for 16 weeks (n = 5). Scale bar, 50 μm. (**K**) Quantification of total cholesterol in the liver of control and Ad-Sept7-KO mice fed a HFD for 16 weeks (n = 3–4). (**L**) Quantification of triglyceride in the liver of control and Ad-Sept7-KO mice fed a HFD for 16 weeks (n = 4). (**M**) Quantification of total cholesterol in serum of control and Ad-Sept7-KO mice fed a HFD for 16 weeks (n = 10). (**N**) Quantification of triglyceride in serum of control and Ad-Sept7-KO mice fed a HFD for 16 weeks (n = 6–8). (**O**) Quantification of HDL-C in serum of control and Ad-Sept7-KO mice fed a HFD for 16 weeks (n = 8). (**P**) Quantification of LDL-C in serum of control and Ad-Sept7-KO mice fed a HFD for 16 weeks (n = 7–8). Shown are mean values ± SEM. ∗P ≤ 0.05; ∗∗P ≤ 0.01; ∗∗∗P ≤ 0.001; ns, not significant.

Our identification of SEPT7 as a negative regulator of adipogenesis while promoting lipolysis provided an opportunity to test whether the augmentation of adiposity in Ad-*Sept7*-KO mice is metabolically disadvantageous. We assessed glucose homeostasis and insulin tolerance in CD and HFD-fed wild-type and Ad-*Sept7*-KO mice. No changes in fasting glucose level, glucose or insulin tolerance were observed in lean CD-fed mice between wild-type and Ad-*Sept7*-KO mice (Figure 4A–D). However, after 8 weeks HFD, Ad-*Sept7*-KO mice developed impaired glucose tolerance. At 16 weeks HFD, the impairment in glucose tolerance was progressed in Ad-*Sept7*-KO mice (Figure 4E–F). On the other hand, HFD-fed Ad-*Sept7*-KO mice showed similar insulin tolerance compared to littermate controls (Figure 4G–H). Furthermore, while HFD-fed Ad-*Sept7*-KO mice showed the characteristic signs of nonalcoholic fatty liver including enlarged liver and increased amount of hepatic lipid deposition (Figure 4I–J). H&E staining revealed that the liver tissues of Ad-*Sept7*-KO mice exhibited an increase in the size of hepatocytes, accompanied by a significant enlargement of intracellular vacuoles, which represent lipid droplet (LD). Additionally, the proportion of hepatocytes containing large vacuoles also increased significantly (Figure 4I). BODIPY staining revealed that Ad-*Sept7*-KO mice exhibited a markedly elevated number of LDs and a significantly larger size of LDs in liver tissue (Figure 4J, Supplementary Figure 5A). We also performed biochemical assays of liver cholesterol and triglyceride (TG) and found increased TG level in HFD-fed Ad-*Sept7*-KO mice (Figure 4K–L). Consistently, HFD-fed Ad-*Sept7*-KO mice showed elevated TG level in the serum compared to wild-type, while the HDL-C and LDL-C levels were similar in two genotypes (Figure 4M–P). Taken together, these data implicated SEPT7 regulates systemic glucose homeostasis in obesity.

### 3.5 SEPT7 negatively regulates expression of PPARγ, C/EBPα while promotes HSL expression in adipocyte

**Figure 5:**
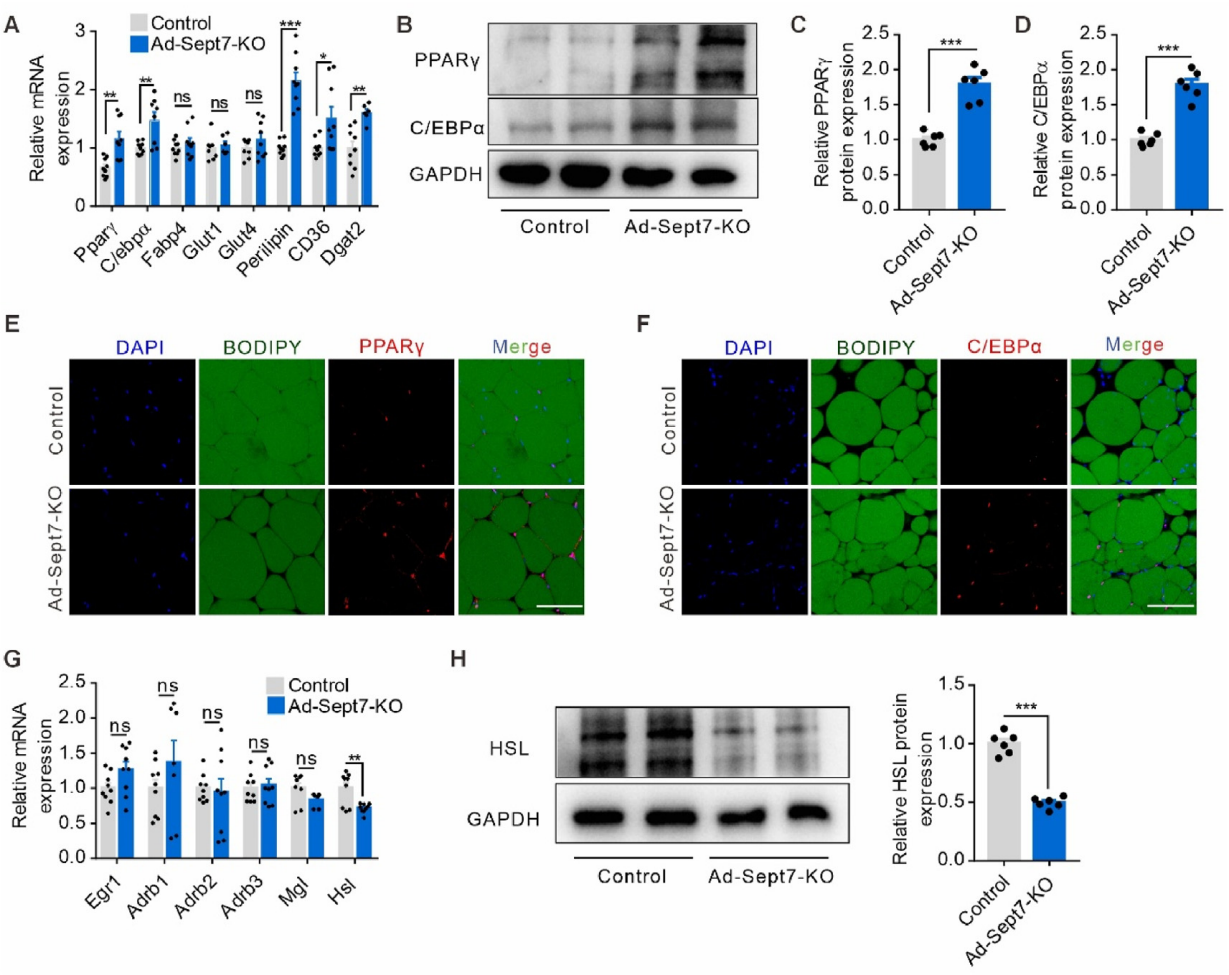
**SEPT7 negatively regulates expression of PPARγ, C/EBPα while promotes HSL**. (**A**) qRT-PCR showing mRNA expression of lipogenesis mediators genes in control and Ad-Sept7-KO mice fed a HFD for 16 weeks (n = 6–9). (**B-D**) Western blot analysis (**B**) and quantification (**C-D**) PPARγ and C/EBPα protein level in white adipocyte isolated from control and Ad-Sept7-KO mice fed a HFD for 16 weeks (n = 6). (**E**) Representative images of immunofluorescence staining for PPARγ (red), BODIPY (green) and DAPI (blue) in adipose tissue isolated from control and Ad-Sept7-KO mice fed a HFD for 16 weeks (n = 5). Scale bar, 50 μm. (**F**) Representative images of immunofluorescence staining for C/EBPα(red), BODIPY (green) and DAPI (blue) in adipose tissue isolated from control and Ad-Sept7-KO mice fed a HFD for 16 weeks (n = 5). Scale bar, 50 μm. (**G**) qRT-PCR showing mRNA expression of lipolysis mediators genes in control and Ad-Sept7-KO mice fed a HFD for 16 weeks (n = 6–9). (**H**) Western blot analysis (left) and quantification (right) HSL protein level in white adipocyte isolated from control and Ad-Sept7-KO mice fed a HFD for 16 weeks (n = 6). Shown are mean values ± SEM. ∗P ≤ 0.05; ∗∗P ≤ 0.01; ∗∗∗P ≤ 0.001; ns, not significant.

**Figure 6:**
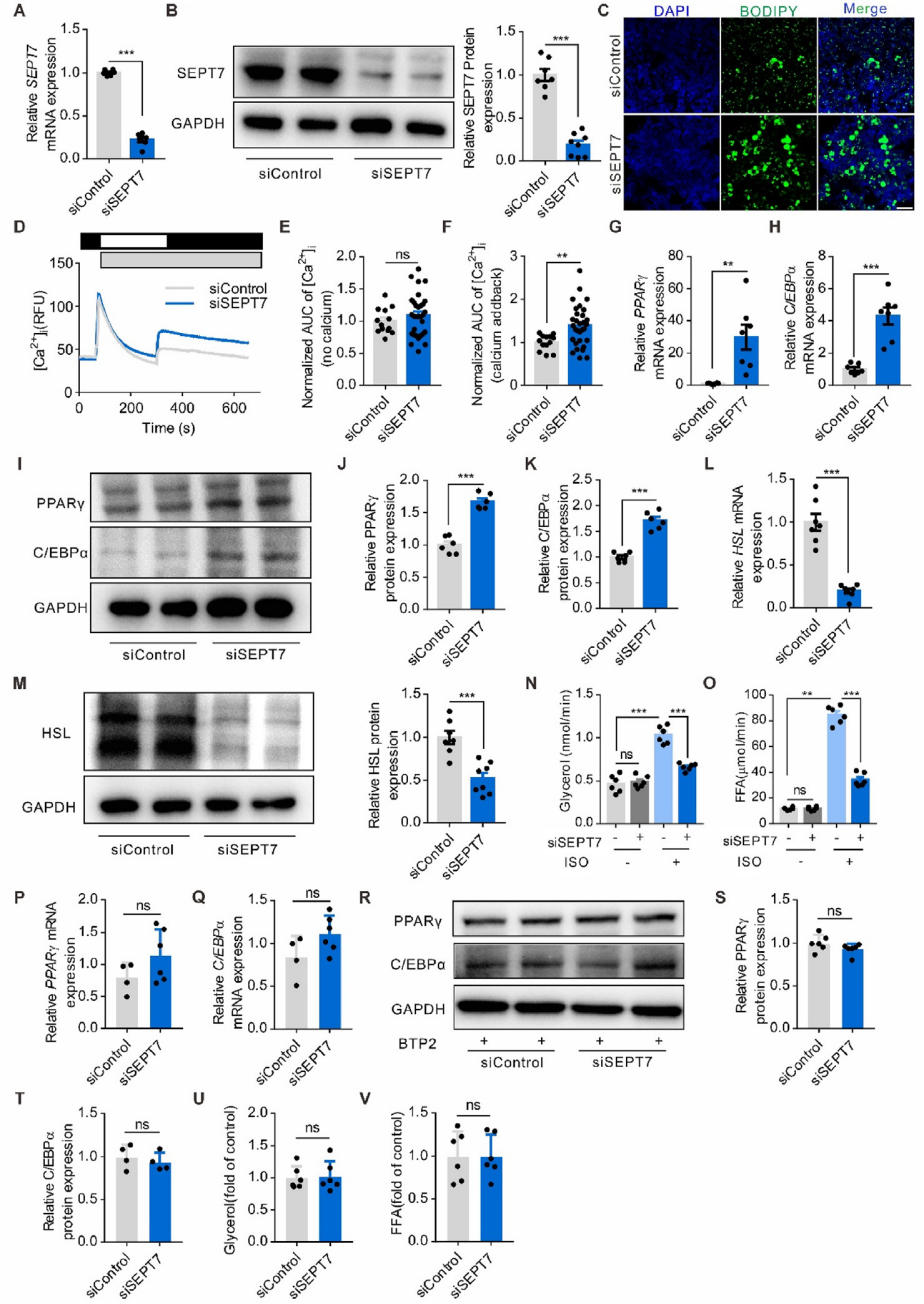
**SEPT7 negatively regulates 3T3-L1 differentiation while promotes lipolysis via PPARγ, C/EBPα and HSL**. (**A**) qRT-PCR showing mRNA expression of SEPT7 in insulin-treated 3T3-L1 cell transfected with SEPT7 siRNA or control siRNA (n = 6). (**B**) Western blot analysis (left) and quantification (right) of SEPT7 protein levels in 3T3-L1 cell transfected with SEPT7 siRNA or control siRNA (n = 7–8). (**C**) Representative images of BODIPY staining of insulin-treated 3T3-L1 cell transfected with SEPT7 siRNA or control siRNA (n = 6). (**D**) SOCE measurement in Fluo-4–loaded 3T3-L1 cells transfected with SEPT7 siRNA or control siRNA (n = 13–31). (**E**) AUC of intracellular Ca^2+^ (no calcium) normalized to the control (n = 13–31). (**F**) AUC of intracellular Ca^2+^ (calcium addback) normalized to the control (n = 13–31). (**G**) qRT-PCR showing mRNA expression of PPARγ in insulin-treated 3T3-L1 cell transfected with SEPT7 siRNA or control siRNA (n = 6–7). (**H**) qRT-PCR showing mRNA expression of C/EBPα in insulin-treated 3T3-L1 cell transfected with SEPT7 siRNA or control siRNA (n = 7). (**I–K**) Western blot analysis (**I**) and quantification (**J-K**) of PPARγ and C/EBPα protein level in insulin-treated 3T3-L1 cell transfected with SEPT7 siRNA or control siRNA (n = 6). (**L**) mRNA expression of HSL in insulin-treated 3T3-L1 cell transfected with SEPT7 siRNA or control siRNA (n = 7). (**M**) Western blot analysis (left) and quantification (right) HSL protein level in insulin-treated 3T3-L1 cell transfected with SEPT7 siRNA or control siRNA (n = 7–8). (**N**) Quantification of glycerol in ISO-treated 3T3-L1 cell transfected with SEPT7 siRNA or control siRNA (n = 6). (**O**) Quantification of FFA in ISO-treated 3T3-L1 cell transfected with SEPT7 siRNA or control siRNA (n = 6). (**P**) qRT-PCR showing mRNA expression of PPARγ in insulin-treated and BTP2-inhibited 3T3-L1 cell transfected with SEPT7 siRNA or control siRNA (n = 4–6). (**Q**) qRT-PCR showing mRNA expression of C/EBPα in insulin-treated and BTP2-inhibited 3T3-L1 cell transfected with SEPT7 siRNA or control siRNA (n = 4–6). (**R-T**) Western blot analysis (**R**) and quantification (**S-T**) of PPARγ and C/EBPα protein level in insulin-treated and BTP2-inhibited 3T3-L1 cell transfected with SEPT7 siRNA or control siRNA (n = 4–6). (**U**) Quantification of glycerol in ISO-treated and BTP2-inhibited 3T3-L1 cell transfected with SEPT7 siRNA or control siRNA (n = 6). (**V**) Quantification of FFA in ISO-treated and BTP2-inhibited 3T3-L1 cell transfected with SEPT7 siRNA or control siRNA (n = 6). Shown are mean values ± SEM. ∗P ≤ 0.05; ∗∗P ≤ 0.01; ∗∗∗P ≤ 0.001; ns, not significant.

To understand the underlying mechanism by which SEPT7 regulates adipocyte metabolism, we performed expression profiling by quantitative RT-PCR (qRT-PCR) using total RNA isolated from WAT of Ad-*Sept7*-KO mice and wild-type littermates, which had been fed HFD for 16 weeks. PPARγ and C/EBPα, master regulators of adipocyte differentiation and fat accumulation, as well as some of their known downstream mediator genes such as Perilipin, CD36, and Dgat2, showed enhanced expression in Ad-*Sept7*-KO mice (Figure 5A). This upregulation of PPARγ and C/EBPα in the absence of SEPT7 was confirmed on the protein level in WAT of HFD-fed Ad-*Sept7*-KO mice and wild-type littermates (Figure 5B–D). Further immunofluorescence staining for PPARγ and C/EBPα showed not only an augmented expression, but also a nuclear enrichment (Figure 5E–F). Lipolysis marker genes expression profiling showed that HSL, a lipase activated by various hormones such as adrenaline, glucagon, thyroid-stimulating hormone and adrenocorticotropic hormone, was down-regulated in WAT of adipocyte-specific SEPT7 deletion mice (Figure 5G). This suppression of HSL in the absence of SEPT7 was confirmed on the protein level in WAT of HFD-fed Ad-*Sept7*-KO mice and wild-type littermates (Figure 5H). To validate those *in vivo* alterations of adipocyte metabolism marker genes, we proceeded to employ siRNA-mediated suppression of SEPT7 on 3T3-L1 cell to assess SEPT7 function in vitro (Figure 6A–B). BODIPY staining showed SEPT7 interference increased the number of 3T3-L1 cells with profoundly larger LD (Figure 6C). Recent study suggested SEPT7 functions as a negative regulator of Ca^2+^ entry by inhibition of SOCE channel [36]. Similarly, SEPT7 knock-down resulted in significantly higher Ca^2+^ entry via SOCE in 3T3-L1 cells (Figure 6D–F). In the meantime, SEPT7 knock-down promoted PPARγ and C/EBPα expression (Figure 6G–K) and suppressed HSL expression (Figure 6L–M) on both transcription and protein level, which is consistent with the *in vivo* results. The ISO-induced elevated glycerol and FFA levels of 3T3-L1 cells were reversed by SEPT7 interference, which indicated that lipolysis was suppressed (Figure 6N–O). However, when SOCE was inhibited by BTP2, the regulatory role of SEPT7 on PPARγ and C/EBPα diminished (Figure 6P–T), and the affected lipolysis activity is reversed as well (Figure 6U–V). Together, these results indicated that SEPT7 restrains SOCE and regulates adipocyte adipogenesis and lipolysis by targeting PPARγ, C/EBPα and HSL.

## 4 Discussion

Septins are guanosine triphosphate-binding proteins that have been retained throughout evolution in all eukaryotic organisms, with the exception of plants [37]. They function as multimeric complexes that interact with membrane lipids, cytoskeleton and microtubules [38]. Based on these interactions, septins play important roles in the physiological functions of many types of mammalian cells, including the regulation of microtubule stability, vesicular transport, cell polarity, and apoptosis [19,39,40]. The introduction section mentions briefly the role of septins in other diseases and health problems, while here the main focus shall be on how septins affect fat metabolism. Gassama-Diagne et al. found that the content and distribution of LDs in HCV-infected hepatocytes correlated with that of lysosomes, and were regulated by oleic acid and SEPT9 [23]. Lou et al. demonstrated that SEPT4 inhibits the formation of LD-rich foam cells, which are associated with the onset and progression of atherosclerosis, via the PPARγ/LXRα pathway [41]. Malagón et al. showed that SEPT11 was upregulated in adipocytes of obese human, and SEPT11 protein levels in omental fat were positively related to serum triacylglycerol, which is similar to our results on SEPT7 [24]. They found that SEPT11 is transcriptionally regulated by insulin and associates with CAV1 and FABP5 at the surface of LDs contributing to lipid traffic. While in current study, we found that SEPT7 restrains SOCE and regulates adipocyte adipogenesis and lipolysis by targeting PPARγ, C/EBPα and HSL [24]. The majority of related studies have concentrated on the relationship between septins and LDs. Given that the envelope of LDs is composed of a single layer of phospholipid molecules, which, together with the lysosomal membrane, represents an extension of the intracellular membrane structure, it is not surprising that their content and distribution are regulated by a cytoskeleton such as the septins [42]. Our study found that the absence of SEPT7 mainly impairs glucose tolerance, with no significant effect on insulin resistance, which is inconsistent with the insulin pathway predicted by bioinformatics analysis by Malagón et al. This may imply that the roles played by various members of the septin family in the regulation of adipocyte metabolism are still nuanced.

As for the potential mechanisms underlying the observed changes in SEPT7 expression in obesity, our research implied a potential anti-obesity role of SEPT7, as its expression increases in response to the obesity. However, at this stage, the molecular mechanism in regulation of SEPT7 during obesity is still largely unknown. This may be because the accumulation of fat causes fat cells to become larger in size, and their cytoskeleton needs to be reprogrammed, which requires more SEPT7 to support the cytoskeleton. Also, Tharp et al. found that adipose tissue utilizes actomyosin machinery to generate tensional responses following adrenergic stimulation, which is another explanation on the alteration of cytoskeleton in responding to obesity [12]. Other studies investigated the microstructure of septins, with a particular focus on the interactions between septins and the various lipid components of the membrane structure, as well as microtubule proteins. Cavini et al. suggested that septins have polybasic regions that allow them to interact with intracellular lipid components [43]. Hu et al. revealed that SEPT7 in *Caenorhabditis elegans* can bind to fat storage-inducible transmembrane protein 2 (FIT2) and enrich the envelope of LDs [44]. This plays an essential role in stabilizing the structure of the LDs and promoting their biosynthesis [44]. SEPT7 is not directly involved in the biochemical reactions of fat metabolism and its function is similar to that of its original scaffold, with the exception that it binds to a new partner, FIT2. The researchers specifically examined the interactions of SEPT2, SEPT6, and SEPT9 with FIT2 and demonstrated that the binding of SEPT7 to FIT2 is one of the most robust within the family [44]. The results demonstrated that the binding of SEPT7 to FIT2 is distinctive within the septin family, indicating that SEPT7 may bind to other uncanonical site rather than fitting in the same spot in the septin complex. This observation provides a novel avenue for research into the potential involvement of SEPT7 in fat metabolism. Our study also addresses the function of SEPT7 in regulating fat metabolism. However, in mouse and human adipocytes, SEPT7 displays properties that are antagonistic to the synthesis of LDs, and instead promotes the growth of LDs with its absence. This indicates that SEPT7 may also possess a FIT2-independent pathway for regulating LD development. Our study observed that SEPT7 negatively regulates PPARγ, which may be the exact mechanism by which SEPT7 regulates the LD maturation. Through the classical synergism, PPARγ and C/EBPα enhance the expression of a series of downstream genes that promote adipogenesis [45]. Those downstream genes include Scd1, Dgat1 & Dgat2, which promote the synthesis of triglycerides, and Glut4, Cd36 & Fabp4, which facilitate the cellular uptake of glucose and fatty acids, and perilipin, which maintains the LD stability [46,47]. In our study, the upregulation of PPARγ and C/EBPα that appeared after adipocyte-specific SEPT7 deletion was observed, accompanied by the upregulation of Perilipin, Cd36, and Dgat2. This result is consistent with the canonical PPARγ pathway. This means that SEPT7 inhibits adipogenesis and intracellular fat deposition by inhibiting the PPARγ pathway.

In our research we found that while male mice exhibited obvious obesity after adipocyte-specific SEPT7 deletion, female mice are much more resistant to it (Supplementary Figure 3). We reviewed the studies on the correlation between SEPT7 and sex or hormones, and some studies pointed out that SEPT7 plays a role in sex determination. Lan et al. found that SEPT7, as a regulatory gene and target gene of miR-202, may affect the growth and development of cattle ovaries [48]. Pan et al. found that SEPT7 is involved in the sex determination of Ussuri catfish, and it mainly plays a role in spermatogenesis [49]. Sreenivasan et al. found that the five members of the septin family are differentially expressed between the gonads and ‘rest-of-body’ of zebrafish, among which SEPT7 had highest expression in the female ‘rest-of-body’ compared to male, which is consistent with our results in mouse adipose tissue [50]. It seems whether SEPT7 is regulated by sex hormones remains to be further studied.

Both GTT and ITT are designed to test a body's capacity to maintain glucose homeostasis. GTT involves ingestion of a glucose solution to evaluate how the body processes glucose, while ITT involves intravenous insulin administration to induce hypoglycemia, assessing the body's response to insulin and counter-regulatory hormone systems [51]. In patients with type-2 diabetes, who suffer from both impaired glucose tolerance and insulin resistance, it is possible to see both GTT and ITT kick back positive results [52]. However, if the glucose tolerance and insulin sensitivity are not damaged in the same time, for example in patients with type-1 diabetes, adrenal insufficiency or pancreatic β-cell dysfunction, these two experiments may present different results [[53], [54], [55]]. In our research, SEPT7-deficient mice on HFD showed positive results on GTT rather than ITT (Figure 4A–H), meaning adipocyte SEPT7-deficiency most likely altered mice's capacity on glucose handling and insulin secretion while their insulin sensitivity or counter-regulatory hormone function were left untouched, which can be observed in metabolic syndrome in some cases [56].

Our study is primarily based on the analysis of bioinformatics data from mouse disease models and the validation on transgenic mouse animal models. The transgenic mice used possess a number of advantageous characteristics, such as an absolute clean deletion, a well-controlled time, and a high level of tissue specificity [57]. Furthermore, the mT/mG fluorescent labelling element has been designed to track the origin of the newborn adipocytes, making it an ideal feature fitting for our study [58]. It is just regrettable that there is a paucity of biological samples based on actual human. In addition to its functions of regulating adipogenesis and lipogenesis, SEPT7 has also been demonstrated to play a role in regulating HSL expression, which in turn affects lipolysis. Given that SEPT7 is upregulated in HFD obese mouse models, it is reasonable to assume that SEPT7 is a protective factor against the further development of obesity induced by a HFD. Further in-depth study is warranted, particularly the analysis of biological samples based on a large number of populations, to clarify whether SEPT7 has a protective effect against obesity as well as other obesity-associated metabolic diseases.

In summary, our study revealed that the deletion of SEPT7 in adipocyte aggregates obesity by promoting white adipocyte differentiation and hypertrophy. This leads to a decreased glucose tolerance, increased hepatic fat accumulation and elevated serum triglyceride. We also provided evidence that SEPT7 restrains SOCE and negatively regulates PPARγ, C/EBPα while promotes HSL expression in WAT. These findings suggest that SEPT7 may represent a novel therapeutic target for the treatment of obesity.

## CRediT authorship contribution statement

**Liran Xu:** Methodology, Investigation, Formal analysis. **Chao Yang:** Writing – original draft, Visualization, Investigation, Formal analysis. **Kaidan Pang:** Methodology, Investigation. **Ying Zhang:** Methodology, Investigation. **Yu He:** Investigation, Funding acquisition. **Siyu Liu:** Investigation. **Huijing Tian:** Investigation. **Zehua Shao:** Investigation. **Siyu Wang:** Investigation. **Xingqian Liu:** Investigation. **Ting Li:** Investigation. **Yapeng Cao:** Investigation. **Luqin Yan:** Validation. **Jinjin Liu:** Validation. **Yanan Wang:** Investigation. **Yongxin Li:** Validation. **Wei Zhao:** Validation, Resources. **Youhua Wang:** Writing – review & editing, Visualization, Resources. **Yang Yan:** Writing – review & editing, Supervision, Resources, Data curation. **Shengpeng Wang:** Writing – review & editing, Supervision, Resources, Project administration, Funding acquisition, Conceptualization.

## Declaration of generative AI and AI-assisted technologies in the writing process

During the preparation of this work the authors used DeepL in order to improve English language and readability. After using this tool/service, the authors reviewed and edited the content as needed and take full responsibility for the content of the published article.

## Declaration of competing interest

The authors declare that they have no known competing financial interests or personal relationships that could have appeared to influence the work reported in this paper.

## Data Availability

Data will be made available on request.
