# Supplementary material for: Adipocyte Septin-7 attenuates obesogenic adipogenesis and promotes lipolysis to prevent obesity
Source: Mol Metab. 2025 Feb 25;95:102114. doi: 10.1016/j.molmet.2025.102114 (PMC11930438; doi:10.1016/j.molmet.2025.102114)
Supplement: Multimedia component 1 [file mmc1.docx]

**APPENDIX A. SUPPLEMENTARY DATA**


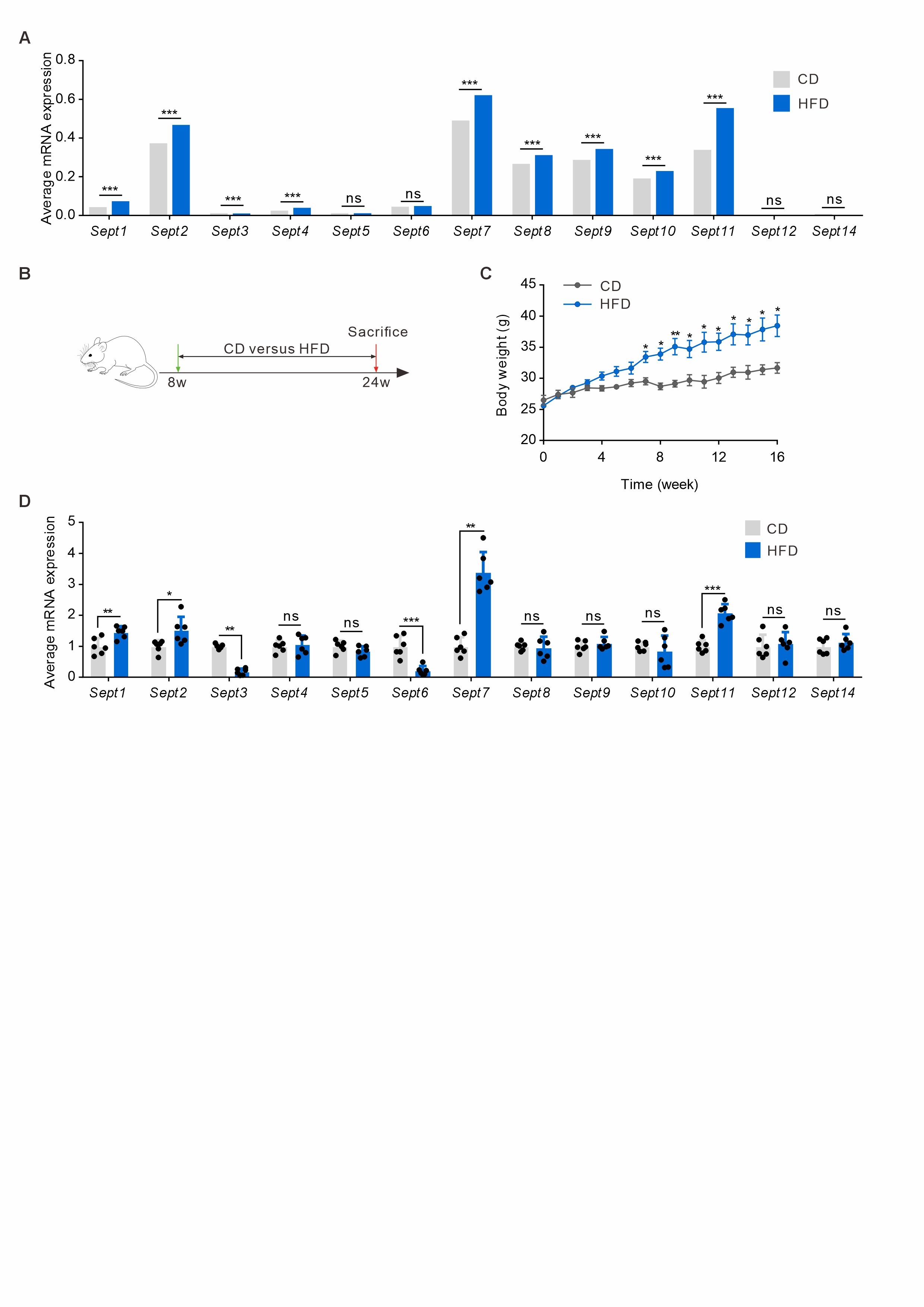


**Supplementary figure 1. SEPT7 is highly expressed in diet-induced obesity mice model.**

(A) Single-cell sequencing data showing SEPT7 is highly expressed in murine adipocytes among the septin family. (B) Schematic diagram of the obese mouse model design. 8-week-old C57 mice were fed with CD or HFD for 16 weeks consecutively and then sacrificed for sample harvest. (C) Body weight of mice fed CD or HFD for 16 weeks (n=5-6). (D) qRT-PCR showing adipose tissue septins expression in mice fed CD or HFD for 16 weeks (n=6). Shown are mean values ± SEM. *P≤0.05; **P≤0.01; ***P≤0.001; ns, not significant.


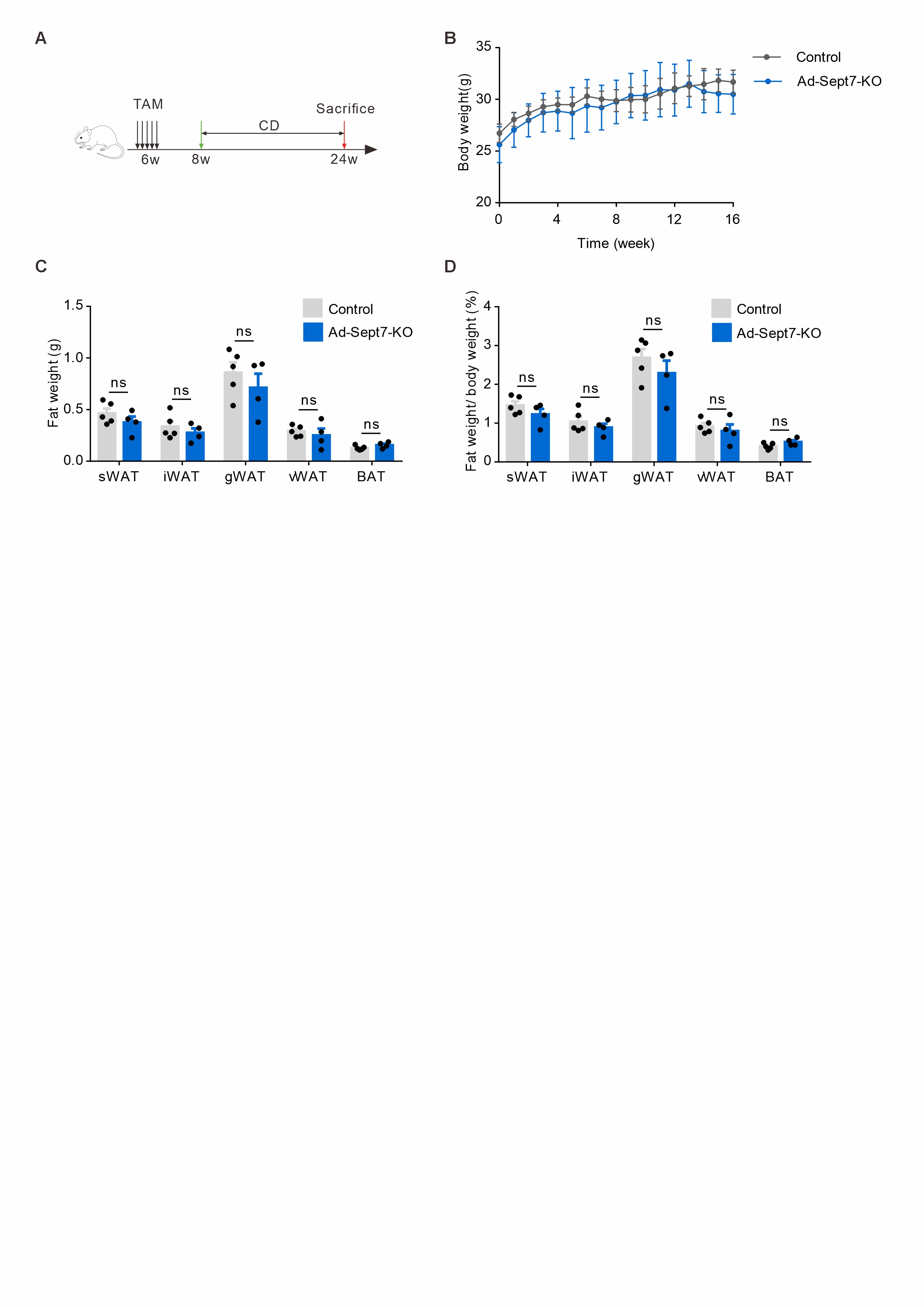


**Supplementary figure 2. Adipocyte-specific SEPT7 deletion does not affect mice body weight and fat weight on CD.**

(**A**) Schematic diagram of the experimental design. Control and Ad-Sept7-KO mice were injected with tamoxifen on 5 consecutive days before 16-week CD. (**B**) Body weight of Control and Ad-Sept7-KO mice fed CD for 16 weeks(n=4-5). (**C**) Various Fat weight from Control and Ad-Sept7-KO mice fed CD for 16 weeks (n=4-5). (**D**) Fat weight/ body weight ratio from Control and Ad-Sept7-KO mice fed CD for 16 weeks (n=4-5). Shown are mean values ± SEM. ns, not significant.


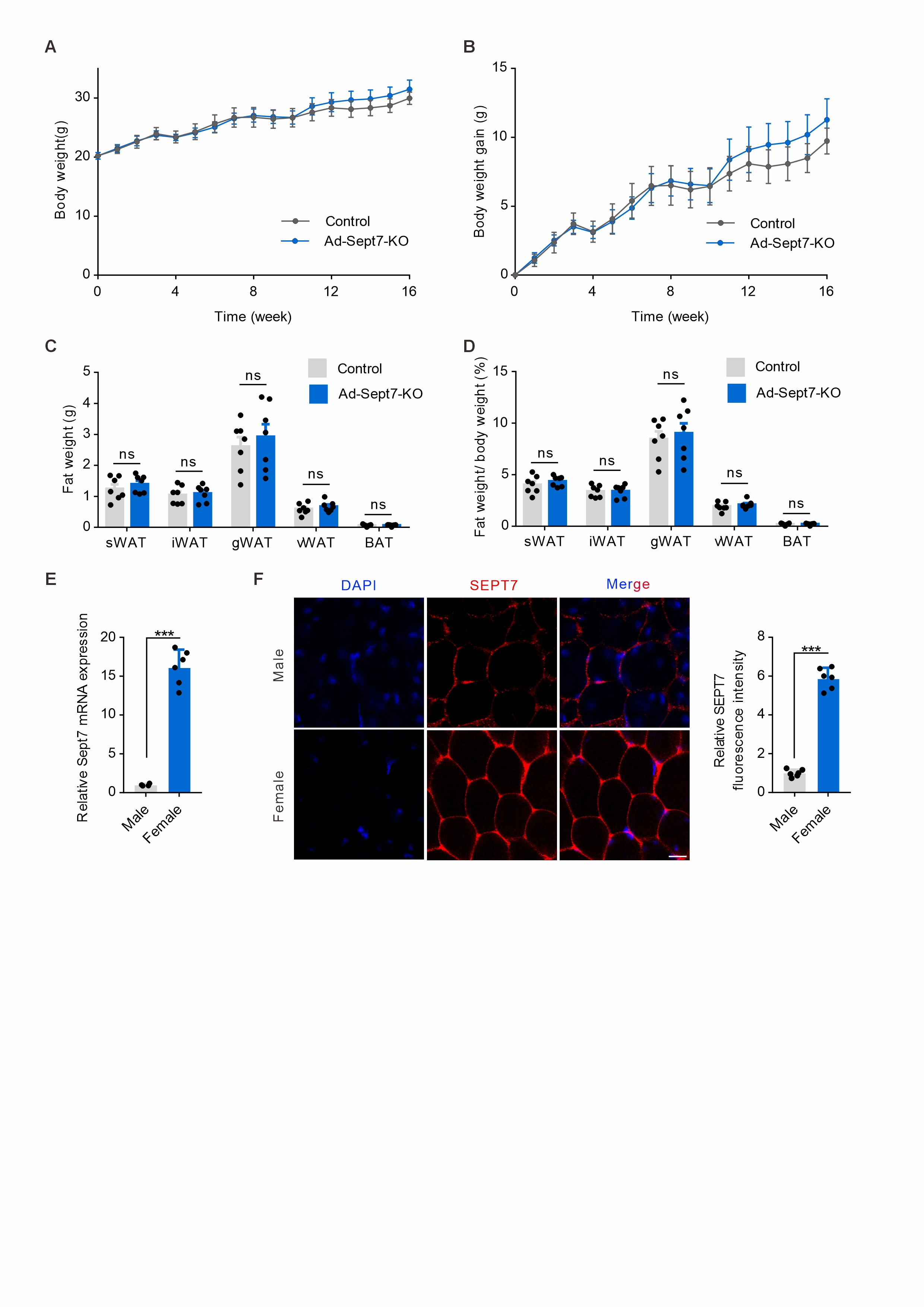


**Supplementary figure 3. The obesity-prompting effects of adipocyte-specific SEPT7 deletion is less decisive in a female only group.**

(**A**) Body weight of Control and Ad-Sept7-KO female mice fed HFD for 16 weeks(n=7-8). (**B**) Body weight gain of Control and Ad-Sept7-KO female mice fed HFD for 16 weeks(n=7-8). (**C**) Various Fat weight from Control and Ad-Sept7-KO female mice fed HFD for 16 weeks (n=7). (**D**) Fat weight/ body weight ratio from Control and Ad-Sept7-KO female mice fed HFD for 16 weeks (n=7). (**E**) qRT-PCR showing mRNA expression of SEPT7 in adipose tissue isolated from male or female C57 mice fed CD for 8 weeks (n=4-6). (**F**) Representative images (left) and quantifications (right) of immunofluorescence staining for SEPT7(red) and DAPI(blue) in adipose tissue isolated from male or female C57 mice fed CD for 8 weeks (n=6). Scale bar, 20μm. Shown are mean values ± SEM. ***P≤0.001; ns, not significant.


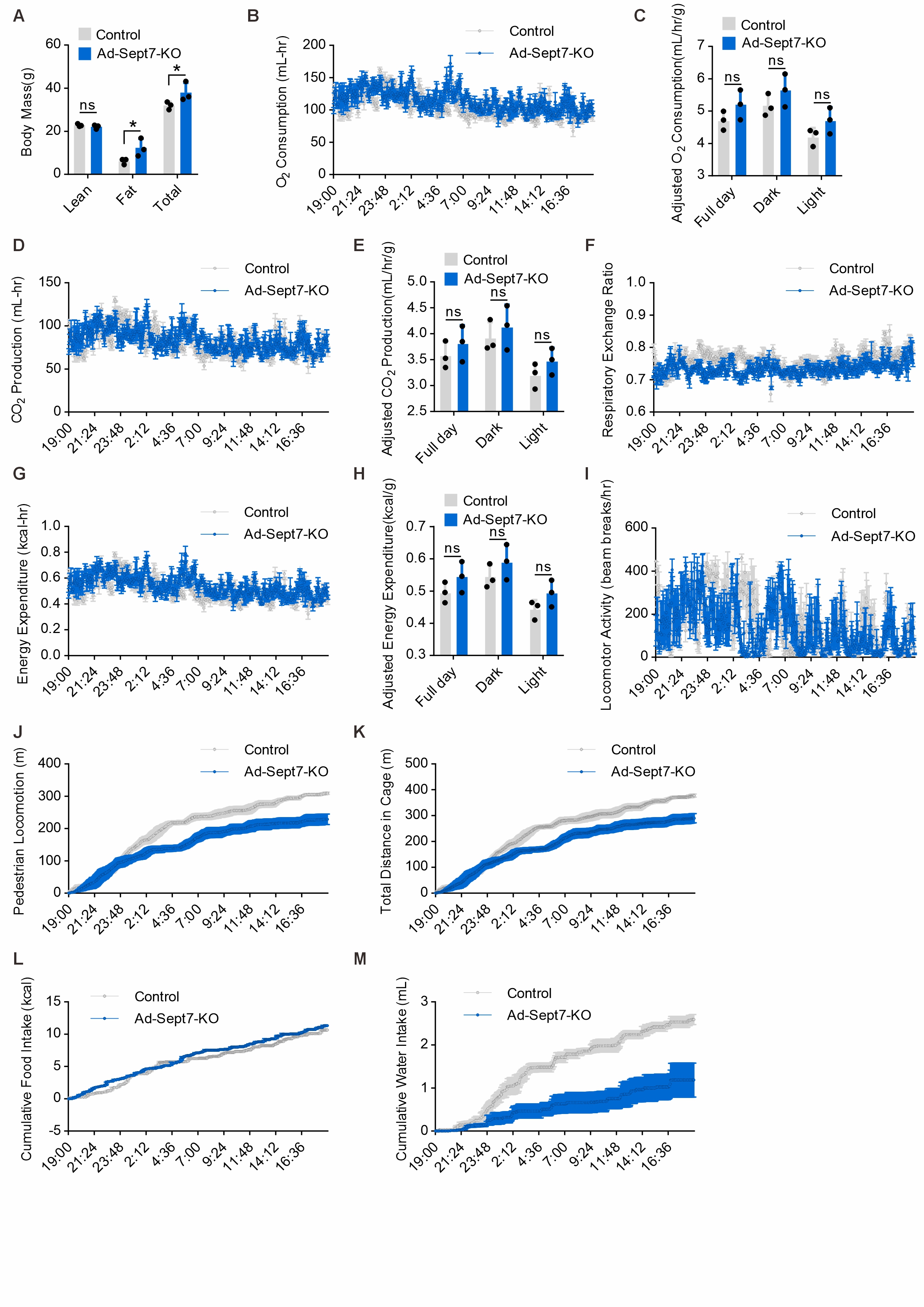


**Supplementary figure4. The effects of adipocyte SEPT7 deletion on the energy expenditure of mice.**

(**A**)Lean mass, fat mass and total body weight of control and Ad-Sept7-KO mice on HFD for 8 weeks (n=3). (**B**) 24-hour oxygen consumption monitor of control and Ad-Sept7-KO mice on HFD for 8 weeks (n=3). (**C**) Full-day, dark-time and light-time oxygen consumption (adjusted for lean mass) of control and Ad-Sept7-KO mice on HFD for 8 weeks (n=3). (**D**) 24-hour carbon dioxide production monitor of control and Ad-Sept7-KO mice on HFD for 8 weeks (n=3). (**E**) Full-day, dark-time and light-time carbon dioxide production (adjusted for lean mass) of control and Ad-Sept7-KO mice on HFD for 8 weeks (n=3). (**F**) 24-hour respiratory exchange ratio monitor of control and Ad-Sept7-KO mice on HFD for 8 weeks (n=3). (**G**) 24-hour energy expenditure monitor of control and Ad-Sept7-KO mice on HFD for 8 weeks (n=3). (**H**) Full-day, dark-time and light-time energy expenditure (adjusted for lean mass) of control and Ad-Sept7-KO mice on HFD for 8 weeks (n=3). (**I**) 24-hour locomotor activity (by beam breaks) monitor of control and Ad-Sept7-KO mice on HFD for 8 weeks (n=3). (**J**) 24-hour cumulated pedestrian locomotion of control and Ad-Sept7-KO mice on HFD for 8 weeks (n=3). (**K**) 24-hour total distance in cage of control and Ad-Sept7-KO mice on HFD for 8 weeks (n=3). (**L**) 24-hour cumulative food intake of control and Ad-Sept7-KO mice on HFD for 8 weeks (n=3). (**M**) 24-hour cumulative water intake of control and Ad-Sept7-KO mice on HFD for 8 weeks (n=3).


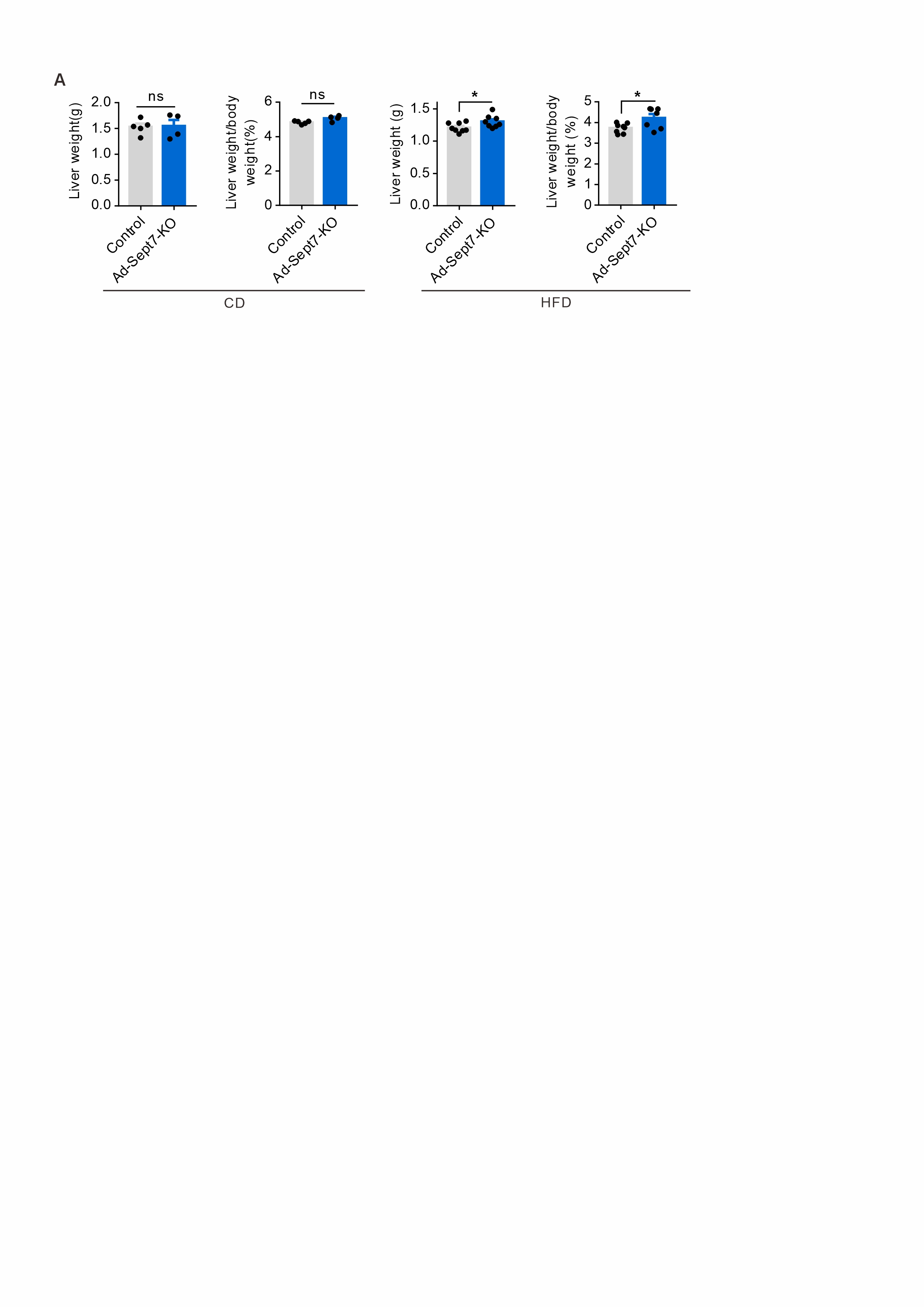


**Supplementary figure 5. The effects of SEPT7 knockout on liver weight and other adipocyte functions related to obesity.**

(**A**) Liver weight and liver/body weight ratio of control and Ad-Sept7-KO mice on CD and HFD for 16 weeks (n=4-8). Shown are mean values ± SEM. *P≤0.05; ns, not significant.
